# Consequences of *CYP2D6* Copy-Number Variation for Pharmacogenomics in Psychiatry

**DOI:** 10.3389/fpsyt.2019.00432

**Published:** 2019-06-20

**Authors:** Joseph P. Jarvis, Arul Prakasam Peter, Jeffrey A. Shaman

**Affiliations:** Coriell Life Sciences, Philadelphia, PA, United States

**Keywords:** personalized medicine, precision medicine, gene deletion, gene duplications, pharmacogenomics, cytochrome P450 CYP2D6, psychiatry, copy-number variation

## Abstract

Pharmacogenomics represents a potentially powerful enhancement to the current standard of care for psychiatric patients. However, a variety of biological and technical challenges must be addressed in order to provide adequate clinical decision support for personalized prescribing and dosing based on genomic data. This is particularly true in the case of *CYP2D6*, a key drug-metabolizing gene, which not only harbors multiple genetic variants known to affect enzyme function but also shows a broad range of copy-number and hybrid alleles in various patient populations. Here, we describe several challenges in the accurate measurement and interpretation of data from the *CYP2D6* locus including the clinical consequences of increased copy number. We discuss best practices for overcoming these challenges and then explore various current and future applications of pharmacogenomic analysis of *CYP2D6* in psychiatry.

## Introduction

### Genetic Variation, Drug Response, and CYP Genes

The clinical application of genomic technologies to enhance prescribing and the customization of pharmaceutical treatment plans is broadly known as pharmacogenomics (PGx). The basic principles of PGx are 1) that genetic variation in key genes involved in the processing and transport of pharmacological agents and their metabolites may alter clinical outcomes in meaningful, patient-specific ways and 2) that similar genetic variation in pharmacological targets may impact an individual’s sensitivity to the effects of particular drugs (1). Though a wide variety of genes have been identified as important players in PGx, the most clinically useful and best studied are members of the cytochrome P450 superfamily. This large group of >50 human genes shows broad similarity in DNA sequence, though members catalyze a variety of reactions. Of clinical importance, several enzymes participate in the phase I detoxification pathway including well-known PGx loci such as *CYP2C9*, *CYP2C19*, and *CYP2D6* (2).

### Single-Nucleotide Polymorphism (SNP) Variation

Over the last two decades, results from published studies in basic science research and clinically oriented journals strongly support the use of PGx in clinical practice (3, 4). Many detail the impact of specific variants, usually single-nucleotide polymorphisms (SNPs), in “CYP” genes on the resulting enzyme’s function. These so-called spelling errors in the genome impart a range of physiological consequences including no change, a measured reduction in protein function, a complete obliteration of function, or even an increase in enzymatic activity. Sometimes, genetic variation involves small insertions or deletions of base pairs instead of the substitution of one base pair for another. This form of variation is known as an “INDEL,” which is a portmanteau of “insertion” and “deletion.” They are often grouped together with substitution mutations such as transitions from A to G or transversions from A to C because they tend to have comparable and often deleterious effects on enzyme function. For most locations in the genome, two copies are inherited—one from the mother and one from the father—that together have the potential to influence patient physiology.

When a patient carries two decreased function alleles, their anticipated physiological state with respect to drug metabolism by that enzyme may be deemed “normal metabolizer,” NM for some genes—including *CYP2D6*—as in the case of a recent CPIC genotype-to-phenotype translation standardization project (5). However, the literature also contains historical interpretations of this combination as “intermediate metabolizer,” IM, or “poor metabolizer,” PM, depending on the particular gene and the specific combination of alleles present (6). Clinically, the reduction or elimination of enzyme function may contribute to an altered response to drug therapy. Depending on the specific pharmaceutical substrate in question, IMs and PMs may fail to clear standard doses of medication or their bioactive metabolites at a normal rate, thus leading to an increased risk of side effects. Alternatively, IMs and PMs may be unable to activate prodrugs such that the patient may fail to fully benefit from the prescribed therapy.

When a patient carries two increased function alleles, or three or more functional gene copies at a given gene of interest, their anticipated physiological state is termed “ultrarapid metabolizer,” UM. When one increased function allele is paired with one normal function allele, overall rates of metabolism are termed “ultrarapid metabolizer,” or the similar “rapid metabolizer,” RM. Clinically, increased metabolism of a drug delivered in its active form may require an increased dose or dividing a higher dose over multiple doses per day in order to achieve consistent therapeutic levels. This is due to the increased metabolism of the drug into its inactive, excretable form. For prodrugs, an alternative therapy or lowered dose may be advisable because a greater-than-usual amount of metabolized, circulating active compound is expected, which may lead to an increased risk of negative side effects (7–9).

### Copy-Number Variants (CNVs)

Another type of genetic change with profound implications for PGx are copy number variants or “CNVs.” In loci that show such variation, two or more copies of the same gene sequence may be inherited from a single parent or the gene may be deleted altogether. Thus, the total number of open reading frames available for the production of fully functional, impaired, or enhanced proteins (depending on the pattern of other variants present), may differ substantially from the expected value of two gene copies. In some cases, the total copy number may be zero or as high as 10 or more (10), which can present in a variety of potential combinations (e.g., five from each parent, six copies from one and four from another, and seven copies from one and three from the other). Clinically, this added dimension of genetic variation can greatly impact the expected physiology for a given set of observations, often introducing significant ambiguity into the process of interpreting patient-specific outcomes (see **Table 1**). For example, when three or more of the duplicated alleles show normal function based on their pattern of SNP and INDEL variants, a state of “ultrarapid metabolism” may occur—a greater amount of functional protein is expected *via* transcription/translation from the greater than two open reading frames. While they may seem to be a somewhat exotic form of variation, CNVs are actually quite common—roughly 12% of the human genome shows some degree of copy-number variation (11), and this includes key PGx genes including *CYP2D6* (12–15). In fact, one study showed that 12.6% of the general US population had copy-number variation in the *CYP2D6* gene (16).

**Table 1:** Potential consequences of duplication for interpreting patient metabolizer status.

Hypothetical metabolizer phenotypes involving non-duplicated alleles			Consequences of duplication			
Metabolizer status	Alleles and activity	Anticipated response	Adding a normal allele	Adding a decreased activity allele	Adding an increased activity allele	Adding a non-functional allele
Normal metabolizer (NM)	Two normal activity alleles, combination of one increased activity allele and one decreased activity allele	Typical metabolism	Increased metabolism	Potentially increased metaboli sm	Increased metabolism	Typical metabolism
Intermediate metabolizer (IM)	One normal activity allele with one non-functional activity allele, two decreased activity alleles	Decreased metabolism	Typical or decreased metabolism	Likely decreased metabolism	Uncertain metabolism	Decreased metabolism
Poor metabolizer (PM)	Only non-functional alleles detected	Little or no metabolism	Decreased metabolism	Likely decreased metabolism	Likely decreased metabolism	Little or no metabolism
Ultrarapid metabolizer (UM)	Two increased activity alleles	Increased metabolism	Increased metabolism	Increased metabolism	Increased metabolism	Increased metabolism

(Left panel) Hypothetical metabolizer phenotypes for specific combinations of non-duplicated alleles with differing activity levels and expected patient physiology, (right panel) predicted changes in metabolizer status when a specific type of additional allele is present (CNV = 3). Ambiguous or uncertain interpretations are shaded red.

### Gene-Conversion Events

Additionally, due to the high degree of sequence similarity among CYP genes, gene-conversion events involving various members including *CYP2D6* have been observed. In a gene-conversion event, a portion of one DNA sequence is effectively pasted over the top of some portion of another’s, creating a hybrid gene containing sequence from both loci. In the case of *CYP2D6*, a wide variety of such hybrid alleles between it and the nearby *CYP2D7* pseudogene have been described (17–21). Clinically, as with many genomic changes, such rearrangements often result in decreased function or non-functional proteins (22).

### Patient Stratification

Regardless of the type(s) of variation involved, many PGx studies include important insights into the way patients that share a particular metabolizer status differ in their response to the therapeutic substrate being studied. However, very few of these studies contain outright and immediately adoptable clinical insights. For example, according to the highly cited PGx knowledge base PharmGKB, only ∼7% of medications have associated genomic information that may be acted upon directly by a physician (1). Interestingly however, these compounds represent ∼18% of all prescriptions written in the US (1). Further, recent reports indicate a large proportion of individuals carry at least one PGx-actionable variant (23) with many bearing two or more. This suggests that the majority of the patient population shows at least some potential to encounter a drug for which PGx information is available. However, the ultimate clinical utility of patient genetic data often depends on assessment and interpretation of the complete combination of variants they possess rather than the presence of one or two variants. So while it is clear that genetic stratification of patients can be a valuable aid to medical practitioners, ordering commercially available PGx reports should be regarded as an enhancement of, rather than a replacement for, current standards of care.

### Potential Benefits of PGx to Psychiatry

**Figure 1:**
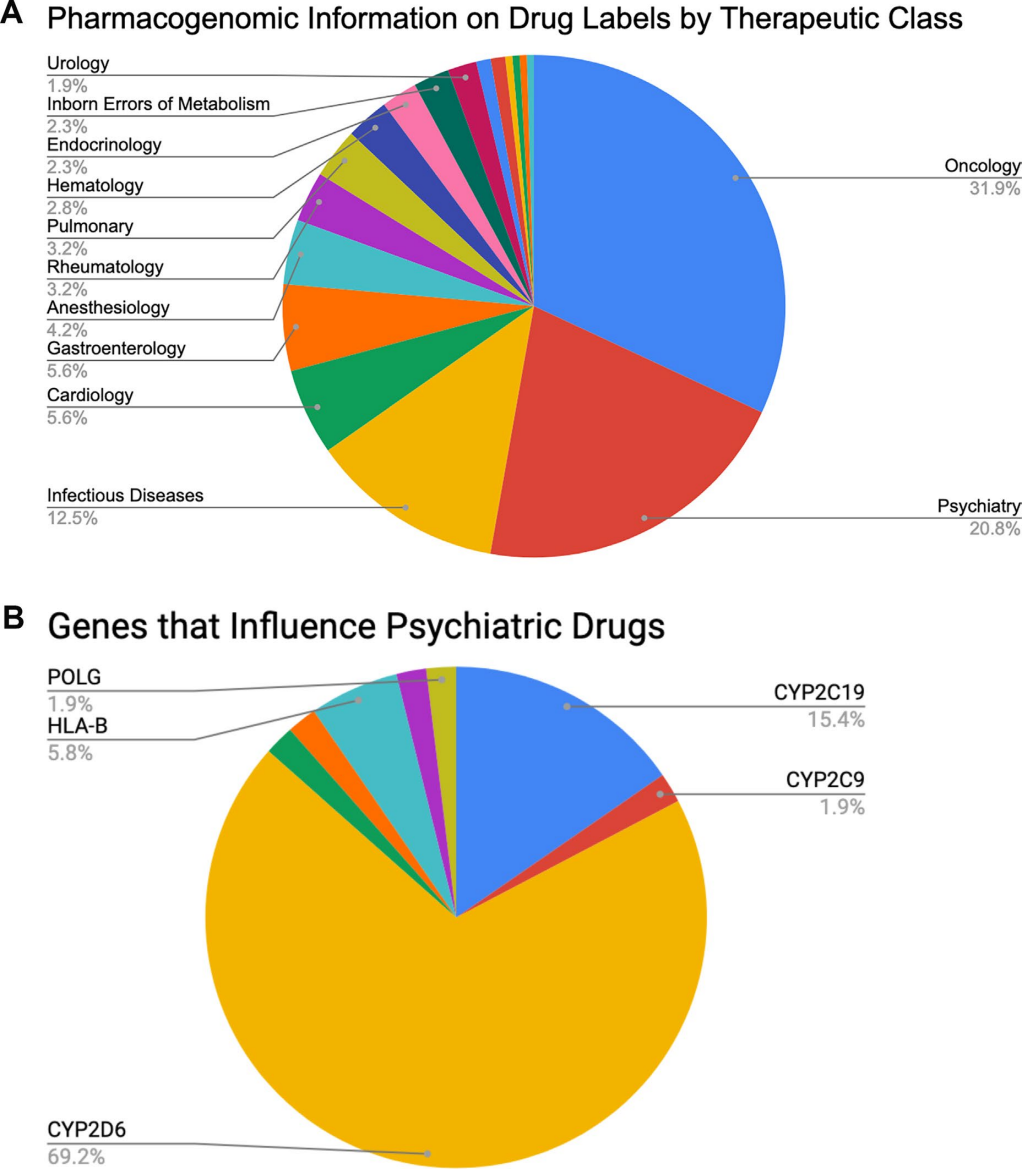
Percentages of **(A)** FDA-approved drugs with available biomarker information in their labels by therapeutic class (total *N* = 215) and **(B)** FDA-approved psychiatric drugs influenced by specific PGx genes of interest (total *N* = 45, see also **Table 2**).

Clinically speaking, the field of psychiatry shows strong potential to disproportionately benefit from the adoption of PGx than do other specialties. Perhaps the most important reason is the relatively high rate of poor clinical outcomes for patients under standard care. For example, between 30% and 50% of psychiatric patients do not respond sufficiently to acute treatment no matter which medication is originally prescribed (24–32), and only 35% to 45% of patients with major depressive disorder return to premorbid levels of function after 6–8 weeks of treatment (24). Thus, there is clearly room for genomic data to inform current clinical practice. Further, psychiatry is the second most commonly observed primary therapeutic area (20.8%, see **Figure 1A**), after oncology (31.9%), on the list of all U.S. Food and Drug Administration (FDA)-approved drugs with available PGx information (33) (summarized in **Table 2**). So, as with oncology, the research literature clearly contains the raw materials for building valuable clinical decision support for psychiatrists. Finally, of those roughly 20.8% of FDA-approved drugs with PGx information that is used in psychiatry, 69.2% are fully processed in some way by a single gene: *CYP2D6* (see **Figure 1B**). This represents a substantial enrichment of CYP2D6 substrates in psychiatric drugs since, overall, CYP2D6 is known to impact the metabolism of ∼25% of all FDA-approved medications. These data suggest an excellent opportunity to focus on a high-value genomic region with great potential for improving patient outcomes. Thus, despite both biological and technical challenges to measuring and interpreting data from *CYP2D6*, the locus may hold the key to important improvements to the standard of care for psychiatric patients.

**Table 2:** The 45 drug products that are FDA-approved for use in neurology and psychiatry that contain pharmacogenomic information in their drug labels, and the gene/biomarker of interest for each. A total of 36 drug labels include actionable or informative pharmacogenomic information in labeling sections Dosage and Administration, Warnings and Precautions, Adverse Reactions, Drug Interactions, Clinical Pharmacology, and Use in Specific Populations (33).

Drug	Biomarker
Amitriptyline ^†^	*CYP2D6*
Aripiprazole ^Rx,Pop,C.Ph^	*CYP2D6*
Aripiprazole Lauroxil ^Rx,Pop,C.Ph^	*CYP2D6*
Atomoxetine ^Rx,!†,ADR,DDI,C.Ph^	*CYP2D6*
Brexpiprazole ^Rx,Pop,C.Ph^	*CYP2D6*
Brivaracetam ^C.Ph^	*CYP2C19*
Carbamazepine ^!,BW,†,!^	*HLA-A, HLA-B*
Cariprazine ^C.Ph^	*CYP2D6*
Citalopram ^Rx,!,C.Ph^	*CYP2C19*
Citalopram ^C.Ph^	*CYP2D6*
Clobazam ^Rx,Pop,C.Ph^	*CYP2C19*
Clomipramine ^†^	*CYP2D6*
Clozapine ^Rx,Pop,C.Ph^	*CYP2D6*
Desipramine ^†^	*CYP2D6*
Desvenlafaxine ^C.Ph^	*CYP2D6*
Deutetrabenazine ^Rx,!†,Pop,C.Ph^	*CYP2D6*
Dextromethorphan and Quinidine ^!†,C.Ph^	*CYP2D6*
Diazepam ^C.Ph^	*CYP2C19*
Doxepin ^C.Ph., C.Ph^	*CYP2D6, CYP2C19*
Duloxetine ^DDI^	*CYP2D6*
Escitalopram ^DDI, ADR^	*CYP2D6, CYP2C19*
Eteplirsen ^I&U,ADR,Pop,Cli^	*DMD*
Fluoxetine ^†,C.Ph^	*CYP2D6*
Fluvoxamine ^DDI^	*CYP2D6*
Galantamine ^C.Ph^	*CYP2D6*
Iloperidone ^Rx,!†,DDI,C.Ph^	*CYP2D6*
Imipramine ^†^	*CYP2D6*
Lacosamide ^C.Ph^	*CYP2C19*
Meclizine ^C.Ph^	*CYP2D6*
Modafinil ^C.Ph^	*CYP2D6*
Nefazodone ^†^	*CYP2D6*
Nortriptyline ^†^	*CYP2D6*
Oxcarbazepine^!†^	*HLA-B*
Paroxetine ^DDI^	*CYP2D6*
Perphenazine ^†,C.Ph^	*CYP2D6*
Phenytoin ^C.Ph., C.Ph.;!^	*CYP2C9, CYP2C19, HLA-B*
Pimozide ^Rx,†^	*CYP2D6*
Protriptyline ^†^	*CYP2D6*
Risperidone ^DDI,C.Ph^	*CYP2D6*
Tetrabenazine ^Rx,!†,Pop,C.Ph^	*CYP2D6*
Thioridazine ^CI,!,†^	*CYP2D6*
Trimipramine ^†^	*CYP2D6*
Valbenazine ^Rx,!†,Pop,C.Ph^	*CYP2D6*
Valproic Acid ^BW,CI,!†, CI,!†^	*POLG*, nonspecific
Venlafaxine ^†^	*CYP2D6*
Vortioxetine ^Rx,C.Ph^	*CYP2D6*

ADR, Adverse Reactions; BW, Boxed Warning; C.Ph., Clinical Pharmacology; CI, Contraindications; Rx, Dosage and Administration; DDI, Drug Interactions; †, Precautions; Pop, Use in Specific Populations; !, Warnings; !†, Warnings and Precautions; I&U, Indications and Usage; Cli, Clinical Studies.

### CYP2D6: Structure, Observed Variation, and Nomenclature

**Figure 2:**
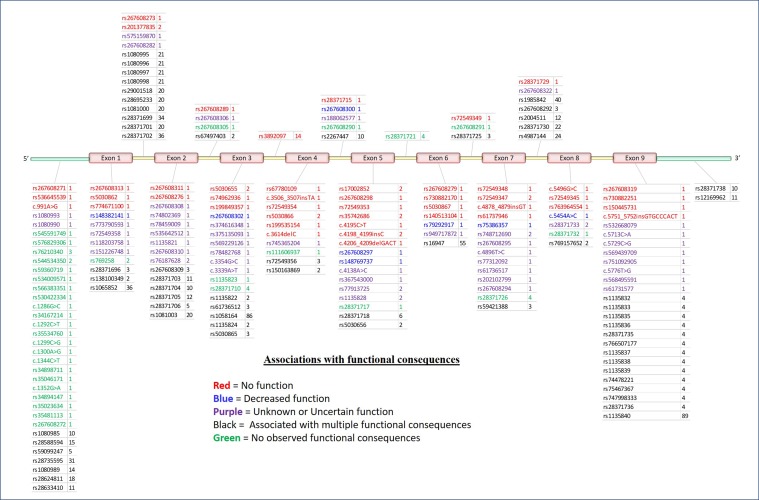
5′ to 3′ structure of the *CYP2D6* locus and placement of variants. Physical position of known pharmacogenomics (PGx) variants in *CYP2D6* by rsID (total *N* = 198). Colors indicate putative functional consequences: red = no function, blue = decreased function, purple = unknown or uncertain function, black = single-nucleotide polymorphism (SNPs) with multiple associated functional consequences [e.g., rs1135840 can be found in alleles with normal function, decreased function, and even non-functional (e.g., *35, *17, and *4, respectively)], green = no observed functional consequences to date (normal). Numbers to the right of each rsID indicate the total number of haplotypes (* alleles) on which each variant is known to be found.

The *CYP2D6* gene consists of nine exons and is found on the negative strand between 42,126,499 and 42,130,881 bp (GRCh38.p12) on chromosome 22q13.2. As shown in **Figure 2**, variation at *CYP2D6* occurs in exons, introns, and both the upstream and downstream regions of the locus.

To date, a total of 198 separate variants of various types have been cataloged for *CYP2D6* (18) (PharmVar version 3.4, **Figure 2A**). In order to arrive at a useful clinical interpretation, the subsets present in any given patient must be considered simultaneously. As such, the concept of the “haplotype”—commonly referred as “* alleles” (read as “star alleles”) in CYP genes—and the related nomenclature standards (22) for *CYP2D6* alleles are critical to understand. Here, “haplotype” refers to the precise combination of variants found on the physical strand of DNA inherited from a specific parent. The combination of the two haplotypes inherited from both parents is collectively known as the “diplotype,” and it is generally the convention to list the lowest numbered haplotype first (34). Ultimately, it is important to remember that * alleles are *CYP2D6* haplotypes that may involve multiple sites and types of variation.

**Figure 3:**
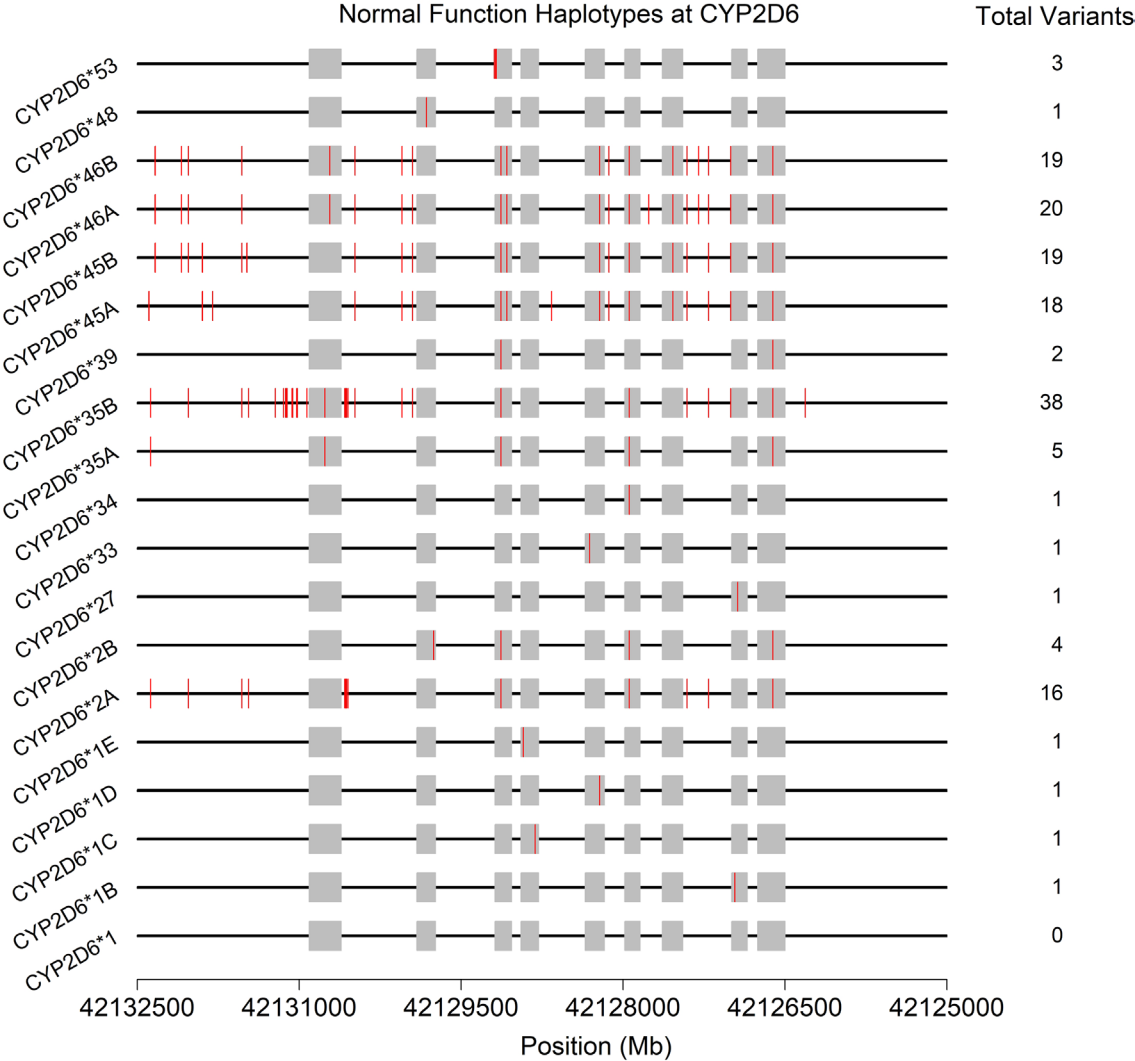
Physical positions (red vertical lines) and total number of variants found on the 19 haplotypes (* alleles) predicted to produce normally functioning enzymes upon transcription/translation. See also **Supplemental Figures 1–4**. Gray boxes indicate the exons (genome build 37).

Often, the same SNP may be found on multiple genetic backgrounds, which, based on the totality of variants present, show different activities (rsIDs in black in **Figure 2**). Further, many named/characterized collections of variants are defined by large numbers of genetic changes (**Figure 3** and **Supplemental Figures 1–4**, see *35B (CYP2D6*35.002), which contains 38 variants, and *2A (CYP2D6*2.001), which bears 16), some of which are shared by multiple named alleles. For example, the C > T variant in exon 1 known as rs1065852 (22:42130692 in GRCh37, c.100C > T, p.Pro34Ser, P34S) is present in at least 36 distinct haplotypes (**Figure 2**). Thus, one important technical challenge for converting raw genetic data into an accurate determination of diplotype is to understand which variants were inherited together from each parent.

Once characterized, each unique haplotype that has been observed is assigned its own “*” designation and is logged in various databases and public resources (35, 36). In the most simple cases, a single variant fully defines a haplotype. For example, the presence of a “C” at position rs5030867 is currently all that is needed to identify a *7 haplotype at *CYP2D6*. In more complicated cases, the simultaneous presence of many types of variation (e.g., SNPs, CNV, and gene-conversion events) defines the haplotypes carried by a given patient. In order to properly identify such complex * alleles, measurements at all—or nearly all—sites of variation are required. When no variation is observed at any tested site, the haplotype is assigned a designation of *1 by default.

When copy-number variation is observed for one or more of the haplotypes, the notation for the duplicated allele is included as “*xN*,” with *N* being the number of copies of the specific allele when it is possible to determine. For example, a CYP2D6 *1x2/*2 diplotype indicates that one allele carries a *1 gene duplication while the other allele carries one *2 gene copy.

### CYP2D6 Haplotyping, SNP Assays, and Clinical Interpretation: Considerations and Challenges

There is an important technical challenge in resolving certain haplotype combinations involving heterozygous variants at multiple locations that does not occur when the observed variation is homozygous. Specifically, if two variants are observed at the same location (e.g., a test result for a specific variant is homozygous in the absence of a whole gene duplication or deletion event), one must have come from one parent and the other from the other parent. Thus, the pattern of inheritance is clear. However, if two variants are observed at different locations, it is unclear whether both variants came from one parent or one variant was inherited from each. The consequences of multi-variant genotypes are particularly complex when they involve changes that completely eliminate enzyme function. Patients showing these combinations have inherited either two non-functional copies of the gene or one normal copy, which is paired with a single non-functional copy carrying both variants. These two possibilities may have very different physiological interpretations depending on the drug involved and consequently different clinical implications. Real-world situations can be substantially more complex to resolve than this simple two-locus example, and novel alleles may also be observed in some patients, which can greatly complicate clinical reporting. Unambiguous ascertainment of the specific distribution of variants on each chromosome yields “phased” haplotypes, something very few of the current technologies are able to produce. Rather, phase is usually estimated using existing knowledge of haplotypes that are expected in the patient genepool and/or *via* mathematical algorithms.

**Figure 4:**
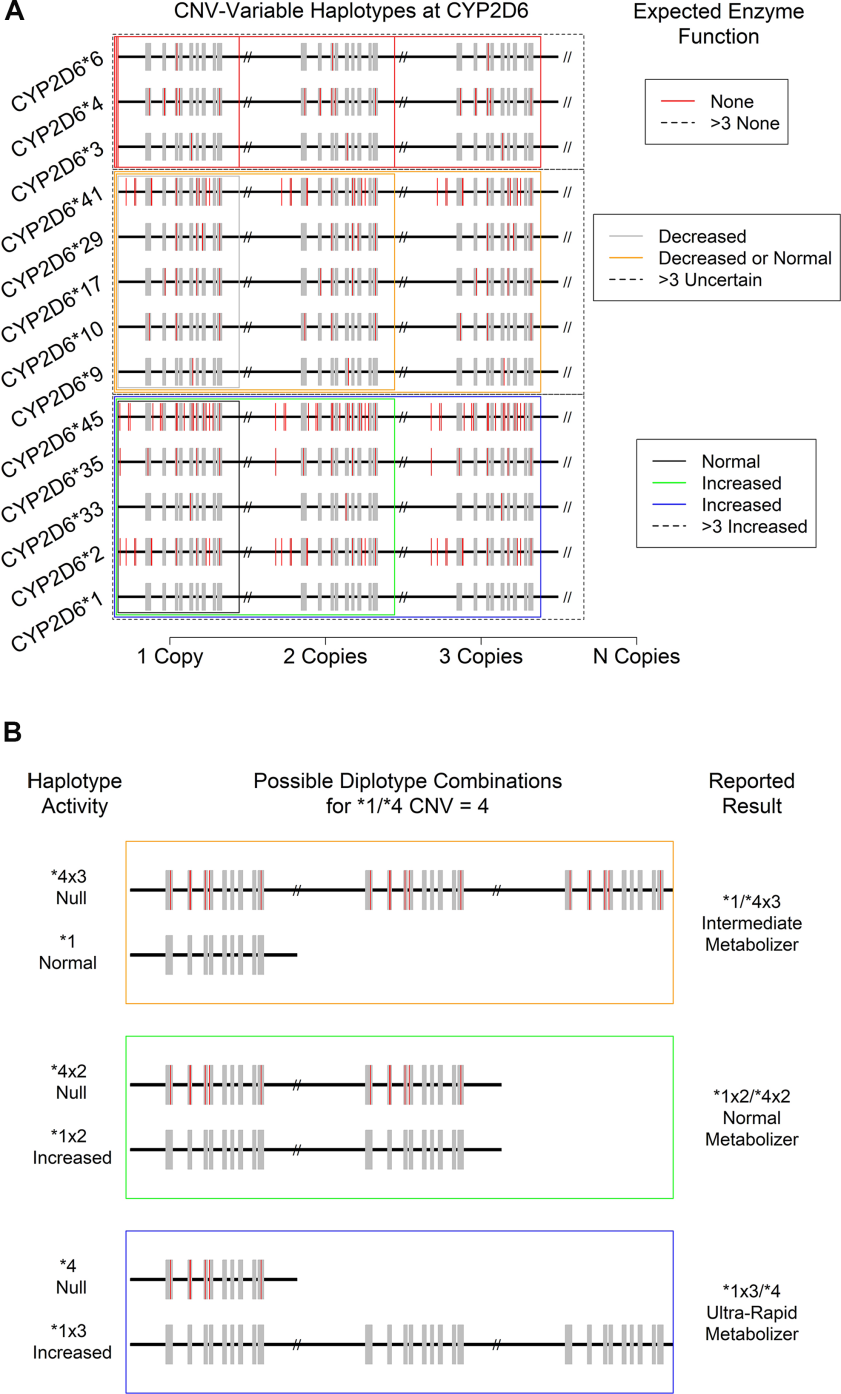
Consequences of *CYP2D6* gene duplication for **(A)** the activity level of 13 expected CNV-variable (duplicated) *CYP2D6* alleles with known enzyme function. Not depicted: CYP2D6 *43, which is also known to show duplications with an uncertain phenotype. **(B)** Individual haplotype (* allele) activity and overall metabolizer status of the “*1/*4, CNV = 4” specific patient result. Note that a copy-number of four introduces ambiguity in the reported metabolizer status due to technical uncertainty regarding which specific allele is duplicated.

At present, the *CYP2D6* haplotypes that confer increased overall function do so *via* increased translation of mRNA to protein due to the presence of two or more gene sequences conferring normal function (CNV-variable haplotypes, see below and **Figure 4**). As such, they must be identified by an assay developed specifically for the purposes of their detection. A similar issue arises with gene conversion polymorphisms that produce hybrid alleles. Since assays for the various types of variation showing measurable consequences for overall CYP2D6 activity in patients are usually performed independently, uniting raw data from all sources into a coherent picture of clinical actionability can be quite challenging. Furthermore, since the effects of specific combinations show a range of physiological effects from non-functional CYP2D6 protein to an increased rate of CYP2D6 enzyme metabolism, clinical interpretation of even accurate genetic profiles can be difficult and are sometimes ambiguous.

It should also be noted that a large number of haplotypes show either unknown—combinations that are too rare or for which there is too little published data to effectively interpret—or, uncertain function—that is, test results/research findings that are conflicting or inconclusive. Diplotypes involving haplotypes with unknown or uncertain functions are particularly difficult to interpret in clinically useful ways, though case studies involving them will be useful in resolving ambiguities. For example, a case study of a non-responding patient who carries a known haplotype with inconsistent evidence in other studies but who clearly benefits from a change in therapy would be a helpful observation and suggests potentially fruitful avenues of future research. These avenues may include *in vivo* phenotyping or pharmacokinetic studies of similar individuals that shed further light on the function of the allele in question.

When patient diplotypes include two haplotypes with clearly defined functions, they may be grouped into potentially substrate-specific metabolizer status groups including normal (NM, previously called extensive, EM), intermediate (IM), poor (PM), and ultrarapid (UM). Depending on a full understanding of clinical and basic science research, the expected physiological consequences of membership in each group may then be developed into clinical decision support.

### CYP2D6: CNV-Variable Haplotypes

The CYP2D6 *5 allele is a complete deletion of the gene sequence that can be inherited from one or both parents. Functionally, as no protein can be produced from the *5 allele, it imparts a complete elimination of CYP2D6 enzymatic function. Therefore, in the physiological interpretation of metabolizer status, it is generally treated in a similar way as other alleles lacking functionality and represents the extreme end of the functional continuum for all substrates. However, there are technical challenges that arise when *5 alleles are present. For example, when *5 is paired with a *1 allele (i.e., no SNP or INDEL variants are observed), technical limitations may cause the patient to appear to carry two normally functioning alleles (*1/*1) until copy-number status is measured (37). Thus, in the absence of CNV data, accurate clinical interpretation of results may not be possible even for examples that appear to be relatively simple from a genetic perspective. Similar complications arise when *5 is paired with alleles carrying various combinations of variants—the technical results will appear as though the patient is homozygous for all observed variants rather than a heterozygous together with a *5 allele. This, in turn, has the potential to introduce considerable ambiguity in clinical interpretation.

Just as there can be wholesale deletions of a *CYP2D6* allele, the chromosomal region where *CYP2D6* is found can carry two or more copies of the *CYP2D6* gene. Such duplications can involve gene units that are functional (e.g., *1xN and *2xN) or non-functional (e.g., *4xN) or those that show decreased function (e.g., *41xN), leading to a variety of potential clinical consequences. To date, 14 haplotypes bearing various combinations of SNPs and INDELs have been observed to be duplicated in one or more individual (14, 38) (**Figure 4A**). The most commonly observed duplicated alleles are *1, *2, and *4 (15). While other duplications appear more rarely, they do occur at appreciable frequencies in clinical populations and thus must be considered when resolving diplotype combinations and reporting their clinical consequences. It should be noted that, since current catalogs of human genomic variation are incomplete, and especially so for populations of non-European origins, other duplications involving known and as-yet-undocumented alleles likely exist somewhere in the human population. As databases and other genomic resources improve in their sampling of globally diverse populations over time, both the accuracy of diplotyping and the accuracy of the associated clinical decision support are expected to improve.

### CYP2D6 CNV Assays: Considerations and Challenges

While the technical sensitivity of laboratory assays for CNVs at *CYP2D6* can vary, some are capable of accurately discerning the total number present up to 5 and even 10 copies. However, many assays can only provide a CNV resolution of >2, and in the majority of cases, resolution becomes less certain at levels >4. Critically, and as noted above, the CNV and SNP/INDEL assays are often performed independently, and available databases/catalogs of variation are incomplete. Thus, it can be difficult to determine which * allele should be assigned which copy-number. For example, for a patient assayed as CYP2D6 *1/*2 with a copy-number of 4, a fully descriptive clinical report should present the results as “*1x3/*2, or *1x2/*2x2, or *1/*2x3,” because the haplotype of the duplicated gene was not determined with certainty. Fortunately, in this case, the physiological interpretation of metabolizer status and thus the clinical consequences are identical for all three possibilities. Specifically, since both *1 and *2 show normal function, each potential result yields the same ultrarapid metabolizer status designation (**Figure 4A**, bottom boxes: black, green, and blue). Likewise, a *CYP2D6* *4/*6 patient with any copy-number value may be interpreted as a PM since no matter how many copies of either allele are present, all protein produced is expected to be non-functional for the specific substrate of interest (**Figure 4A**, top red boxes).

The situation becomes increasingly complex when duplicated alleles with *different* functional characteristics are present. For example, in a *CYP2D6* *1/*4 patient with CNV = 4 (**Figure 4B**), there are again three distinct possibilities for their diplotype: *1x3/*4, *1x2/*4x2, and *1/*4x3. However, in this case, each is associated with a distinct metabolizer status (UM, NM, and IM, respectively), and so each may have a different clinical interpretation (e.g., increased dose, standard dose, or decreased dose of a drug delivered in its active form). Such ambiguous results should be interpreted with caution and in concert with the specific patient’s medical and drug response history (if available). Previous adverse drug reactions and past medication efficacy may or may not shed light on the actual diplotype and metabolizer status present. This combination of genetic testing and traditional clinical approaches to treatment likely represents a best-case scenario for certain genetically complex results.

### CYP2D6 Ultrarapid Metabolizers in Clinical Practice

The measurement of total copy-number at the *CYP2D6* locus is particularly crucial for PGx in clinical psychiatry. Currently, the only known way for CYP2D6 metabolism to be increased is *via* duplication of one or more of the CNV-variable * alleles with normal or decreased function. Further, the most useful PGx-based clinical decision support for many of the drugs used in psychiatry can be provided for ultrarapid metabolizers, which, by definition, must carry one or more duplicated allele.

While there are no overarching guidelines for the use of metabolizer status in clinical practice, strong evidence-based research and outcomes data support their utility in many contexts. For instance, the US FDA’s Center for Drug Evaluation and Research allows for and approves the addition of metabolic status and dosing impacts and warnings directly to drug labels. Similarly, independent pharmacogenomic consortia have included actionable PGx information in guidelines. For example, the Dutch Pharmacogenetics Working Group (DPWG) has 47 guidelines (39), and the Clinical Pharmacogenetics Implementation Consortium (CPIC) has 19 peer-reviewed and published guidelines on 40 gene–drug pairs (40). These guidelines commonly highlight ultrarapid metabolism at CYP2D6 as important for the care of patients.

Indeed, it has long been known that additional functional copies of *CYP2D6* impact the pharmacokinetics of various substrates including nortriptyline and debrisoquine (38, 41). It was shown that elimination rates of nortriptyline were five-fold higher than those of PMs for subjects carrying just a single additional copy of *CYP2D6*, that is, three total copies (38). When 13 functional copies were present, the rate was 17-fold higher than for subjects with no active CYP2D6 enzyme. Further, it was subsequently shown that quinidine inhibition of debrisoquine metabolism in individuals carrying 3, 4, or 13 normal copies of *CYP2D6* could be used to potentially alter clinical outcomes. These data together suggest that advance knowledge of a patient’s metabolizer status at CYP2D6 *via* genetic testing could be invaluable in avoiding issues such as treatment resistance and/or toxicity (42).

Similar information has the potential to greatly inform the choice of therapy and dosage in multiple contexts since CYP2D6 processes several clinically valuable anxiolytics, antidepressants, and antipsychotics (see **Table 2**). For example, a meta-analysis reports that the dosage of ∼50% of commonly used antipsychotics is dependent on *CYP2D6* genotype (24). In addition, extraordinarily high clearance rates of the antidepressant trimipramine have been observed in three carriers of duplications at *CYP2D6* taken from a group of healthy subjects. Ultimately, the authors suggest that a dose of up to 200% of average may be required for such individuals to attain similar concentrations as normal metabolizers (43). Further, since CYP2D6 is responsible for the hydroxylation of trimipramine and not its methylation (44), the effects of CYP2D6 UM status on the active metabolite desmethyltrimipramine must be considered as well since it is also metabolized by CYP2D6. Thus, the potential difference in clinical effects may be even larger than considering trimipramine alone (43).

The same study explored the effects of duplication at *CYP2D6* on the tricyclic antidepressant doxepin in healthy subjects. The authors observed that ultrarapid metabolizers showed levels of the active metabolite *N*-desmethyldoxepin at 40% of those seen in normal metabolizers, and considering both this active daughter compound and levels of doxepin itself, NMs showed levels two-fold higher than did UMs. Similarly, interpretable trends are seen for the tricyclic antidepressants imipramine and desipramine (10, 45).

In a retrospective study of non-responsiveness to antidepressants metabolized by CYP2D6, a complete absence of UMs was observed in the subset of patients (*N* = 28) experiencing adverse drug events (ADEs), while eight were identified as PMs. The authors conclude that this enrichment is four-fold higher than expected by chance alone. Conversely, in a subset of 16 non-responders without ADE, no UMs and only one PM were observed (10). While admittedly a small sample size, these trends strongly support the role of *CYP2D6* duplications in important clinical outcome measures. Finally, in a study that identified 81 non-responders to antidepressants metabolized by CYP2D6, 10% (eight subjects) carried duplications. The authors point out that this is a substantial enrichment over the 0.8–1.0% incidence expected for Nordic Caucasians and that the worst week scores of the Hamilton Depression Rating Scale were greater in those carrying duplicated alleles than in those who did not (10, 46).

The clearance of the *S*(+) form of mirtazapine shows a similar pattern across metabolizer groups and has been observed to be 1.6-fold higher in CYP2D6 UMs than in NMs (47). Since the *R*(−) form is not metabolized by CYP2D6 (48, 49) but does appear to be responsible for cardiovascular ADEs, UMs may be at higher risk for both therapeutic failure and side effects when prescribed high doses of mirtazapine (10, 47). Additionally, therapeutic failure due to the effects of increased metabolism by CYP2D6 can increase suicidal behavior in depression patients (50–52). Moreover, UMs have been found to have an elevated risk of high scores on one of the Hamilton Depression Rating Scales that measures suicidality among unipolar or bipolar depressive inpatients (53). UMs also may show low plasma concentrations of fluoxetine or amitriptyline in monotherapy than do PMs, IMs, and NMs if starting concentrations are at the low end of the range when treating major depressive disorder (54).

In one of the largest long-term patient-blinded randomized controlled trials [Genomics Used to Improve DEpression Decisions (GUIDED)], consisting of 1,167 outpatients diagnosed with major depressive disorder (MDD) and patient- or clinician-reported inadequate response to at least one antidepressant, it was found that treating with pharmacogenetic testing-guided therapy, when compared with treatment as usual, at week 8 showed statistically significant improvements in response (26.0% versus 19.9%) and remission (15.3% versus 10.1%). These results further support the potential role of pharmacogenomic testing in the guided treatment of difficult-to-treat psychiatric patients and the improvement of response and remission rates (55).

Ultrarapid metabolism by CYP2D6 has also been suggested to interact with other genetic factors to influence treatment response in certain patient groups. For example, a recent paper suggests that venlafaxine-XR remission is more common in patients with major depressive disorder who 1) failed to respond to citalopram/escitalopram, 2) had CYP2D6 ultrarapid metabolism, and 3) carried certain allele combinations at *SLC6A4* and *SLC6A2* (56). This potentially greater level of genetic resolution for clinical decision support suggests that finer and finer levels of specificity for specific patient groups may be possible in the future. In some cases, this may involve not only interactions attributable to metabolism of compounds by multiple genes at a particular stage of processing but also the action of the same gene at different stages of detoxification.

## Discussion

### Biology, Technology, Interpretations, and Clinical Decision Support

The *CYP2D6* locus shows a highly complex pattern of genetic variants that are inherited in a multitude of combinations. The effect of any given combination of variants on the translated protein can also vary considerably. In turn, clinical outcomes measured in patients carrying similarly functioning alleles also show a degree of variability, but also enough statistical consistency to show great promise for adding new insights to patient care and the enhancement of standard practice. In order to reap these benefits, however, the technical challenges associated with accurately capturing and interpreting raw, laboratory-derived data must be overcome by those who endeavor to provide clinical decision support based upon it. These include 1) accurately producing data for each SNP/INDEL, gene conversion, and copy-number variant, 2) arranging them into likely haplotypes, 3) inferring the metabolizer status that each combination is likely to impart, and 4) accurately connecting each status to the very latest clinical safety and efficacy information in the ever-evolving landscape of the primary literature. The final step, the burden of the health care provider, is of course integrating this information into treatment plans in ways that benefit patients in clinical scenarios.

Accurately assaying CNVs at the *CYP2D6* locus is particularly critical to ensure maximal clinical benefits of testing. Without this key piece of information, very little confidence can be ascribed to results in many cases. Unfortunately, while FDA-approved methods for assaying variation at *CYP2D6* (e.g., AmpliChip CYP450 from Roche and xTAG *CYP2D6* kit from Luminex) take CNVs into account, some laboratory-developed tests (LDTs) do not (57). Indeed, a somewhat alarming recent publication examining PGx reports noted that nearly a third of those laboratories surveyed appear to have failed to incorporate CNVs into *CYP2D6* testing (58). Furthermore, since methods to measure SNPs and INDELs are usually performed independently from techniques to measure CNVs, nearly all tests are limited in their ability to merge these multiple sources of data into definitive haplotypes (i.e., by unequivocally assigning all variants to specific copies of the gene) (15, 59). As we have also seen, the presence of an allelic deletion (the *CYP2D6* *5 allele) produces complications from both a technical and reporting perspective. However, while current laboratory methods are likely imperfect, they nonetheless produce clinically useful insights overall with strong potential to support the creation of patient-specific treatment plans. In fact, it has been estimated that the costs to treat “extreme” metabolizers at CYP2D6 (either PMs or UMs), compared with NMs, can be as high as $4,000 to $6,000 more per year (60, 61), and so advance knowledge of patient metabolizer status may help both reduce costs and increase the quality of care.

### Best Practices

Ultimately, there are several recommendations for best practices for the use of PGx in psychiatry. First, as with any clinical assay, it is important to choose clinical pharmacogenomic tests that are performed in a Clinical Laboratory Improvement Amendments (CLIA) accredited laboratory. These are laboratories that perform human, clinical testing rather than genetic testing for research purposes. They are required to adhere to an established set of rules and regulations and are audited on a regular basis. Second, choose an assay that measures a reasonable subset of *CYP2D6* SNPs, INDELs, and CNVs. Assays performed to detect SNP/INDEL variation should be capable of capturing the key * alleles that are appropriate to the relevant patient population. With respect to CNVs, assays should be capable of discerning quantitative differences in total allele number between 0 and 5 copies at a minimum. Third, for reporting and interpretation purposes, the effects of all measured variation on enzyme function should be considered simultaneously as haplotypes rather than one variant at a time. This means that CYP450 results should be reported as diplotypes (pairs of * alleles) rather than the presence and/or absence of specific individual variants alone. Further, all possible results from ambiguous combinations should be reported when they arise (e.g., *1x3/*2, or *1x2/*2x2, or *1/*2x3).

Finally, when reviewing results, findings should be placed in their proper clinical context and appreciated for the clinical utility that they may or may not represent. For example, results such as “*CYP2D6* *1/*1, CNV = 2” (i.e., no observed variation by the assay in question) are not simply “non results” but rather indicate that standard precautions and procedures are appropriate with the acknowledgement that unmeasured variants may be present, a clinically useful insight. Further, reports showing ambiguous findings—in terms of the so-called diplotypes, metabolizer status, or the potential clinical impact of the observed variants in a given patient—may not provide clear clinical actions but may be useful in the context of patient history and other clinical factors in the determination of medication therapy management. For example, genetics may indicate that a patient may be either a UM or an NM at CYP2D6, but adverse reactions in their medical record may strongly suggest that one status is more likely than the other. Such cases represent important case studies that should be considered for publication. Other unique clinical scenarios also exist and complicate the interpretation of genetic results. For example, liver transplant recipients are expected to have complex medication processing profiles that resemble the metabolizer status of the donor rather than status indicated by the patient’s own DNA. Clearly, such factors are beyond the scope of laboratory testing based on blood, saliva, or cheek swaps, and thus the responsibility falls to the provider to integrate all relevant information into an overall picture of patient care.

### Clinical Significance of Population Effects and *CYP2D6* *1/*1

The frequencies of certain variants, including some key duplicated alleles, show non-trivial levels of variation across global populations (62). There are multiple clinically relevant effects of this observation. The first and most important is that the *1 designation is currently most appropriate for populations of European origin and may be seriously misleading for individuals with recent ancestry from other continental populations. Current genomic databases of all types, including those widely used for PGx assay development, severely under-represent global genomic diversity. Thus, many SNPs, INDELs, and CNVs with potentially important effects on enzyme function and clinical outcomes are simply not cataloged and so do not appear in laboratory-developed *CYP2D6* assays. As such, until knowledge bases and other resources are more complete, a certain degree of caution should be exercised when interpreting *1/*1 results.

Further, allele frequency differences across continental populations are likely to affect a wide variety of loci genome-wide. Since Phase I detoxification is a complicated process, involving multiple enzymes, that is integrated with many other biological systems, some amount of variation in the effects of PGx loci is expected. For example, if processing by CYP2D6 is generally the rate-limiting step in the metabolism of a particular drug, but another locus is responsible for the processing of a bio-active metabolite, changes in allele frequencies at the second locus may well alter the clinically observable effects of the measured *CYP2D6* variants. Thus, any given patient of average European lineage may show a larger or smaller effect size attributable to *CYP2D6* for a given combination of variants than is expected based on potentially measurable variation at other loci contributing to overall patient physiology.

### The Future of PGx Testing and Clinical Guidance

Despite acknowledged limitations, PGx testing is clearly clinically useful now. This is especially true in psychiatric care. As an enhancement to current practice and an important source of insight into patient physiology and expected drug response, PGx can help ensure maximally effective and minimally risky treatment plans, improve patient outcomes, and contribute to much-needed efficiency in health care spending. Interestingly, these benefits are only expected to increase given 1) the large volume of quality research being published annually, 2) the maintenance and curation of critical knowledge bases focused on aggregating key findings, 3) the development of multiple commercial products ensuring both academic and industry engagement in the field, 4) the growing adoption of PGx in clinical practice, and 5) the ultimate emergence of clear practice guidelines. The broader application of PGx and of prescription decision support tools (63) in routine practice is especially important as it will provide invaluable opportunities to define and refine fruitful hypotheses and targeted research initiatives that directly connect research efforts to endpoints and outcomes of clear clinical importance. More specifically, the evolving utility and expansion in the scope of PGx will be facilitated over the near-term by developments in three key areas: 1) surmounting current technological limitations, 2) the need for clear outcomes data for each medication/diplotype combination, and 3) leveraging PGx research and associated databases to study the genetic influence on endogenous compound metabolism and xenobiotics more generally.

As explained above, there is currently no single technology that can accurately, efficiently, and simultaneously assay all critical types of genetic variation and unequivocally connect them to the specific DNA molecule inherited from a specific parent. This includes most strategies for whole genome sequencing, which also cannot reliably produce this sort of “fully phased” genomic data. However, there are promising techniques in development that are beyond the scope of this review to explore in detail such as long-read, single-molecule sequencing methods (64) that potentially offer this level of genomic resolution. Further, the ability to unambiguously determine the location of every variant across every copy of the gene present in a specific patient would have a profound impact on our understanding of the clinical impact of CNV-variable haplotypes. Once the challenges associated with these approaches are addressed, it will be possible to examine the full picture of genomic variation at key loci such as *CYP2D6* in a patient-by-patient fashion and thus more accurately place them in clinically relevant groupings according to expected enzyme function. Coupled with improved physiology testing approaches to understanding the detoxification process, this enhanced level of detail will undoubtedly uncover specific subsets of patients who will disproportionately benefit or who are at disproportionate risk during the application of particular therapeutic strategies.

However, this appropriate phenotypic grouping of patients (e.g., by metabolizer status) also relies on further refinements in our understanding of the clinical impact of specific, potentially rare combinations of variants. As such, it will also be critical to the future development of PGx reporting that key outcomes data continue to be pursued in multiple populations of interest on a diplotype-by-diplotype basis. The enhancement to our wider understanding of the utility of genomic variation provided by such efforts will undoubtedly allow the transition of PGx interpretations from “informative” on current FDA drug labels to “actionable.” It will also increase confidence in reporting for populations that are understudied at present and likely allow more detailed dosing information (such as those available for aripiprazole) to be available for larger number of pharmaceuticals. Ideally, this work would be performed in large samples of human patients, though this may not be fully feasible due to practical limitations including the very large number of potential confounding variables. Alternatively, in exploring and establishing the “true” gradations of decreased function between non-functional and normal, it may be advantageous to augment human studies with work in cell culture or model organisms in order to achieve sufficient sample sizes and statistical support for physiologically relevant findings.

Finally, the expansion in scope of PGx from the genetics of processing/transport and clinical effects of pharmaceutically marketed compounds into other areas of biochemistry and pharmacokinetics is also clearly on the horizon. For example, CYP2D6 has been identified as a potential metabolizer of endogenous neuroactive substrates (65), suggesting future applications of accurate data from *CYP2D6* in psychiatric care involving internal homeostatic processes/physiology in the “normal” range and natural disease progression. Further, the genetic architecture responsible for processing commercially produced compounds is the very same that handles xenobiotic metabolism more generally. As such, the re-deployment of genome-wide PGx data in service of understanding the consequences of unintentional environmental exposures to chemicals such as perfluoroalkyl and polyfluoroalkyl substances (PFAS) and many other likely neurotoxins is likely to emerge as an important contributor to the future health and wellness of the general population.

## Conclusions

The use of PGx as an enhancement to the current standard of care for psychiatric patients shows great potential to guide therapy and improve outcomes in a wide variety of clinical contexts. However, it should not be viewed as a panacea. Important limitations, both technical and biological, must be kept in mind, and PGx information should be carefully integrated with other patient-specific data in the development of customized treatment plans. Some of these limitations will continue to produce ambiguous results for some patients for the foreseeable future at loci like *CYP2D6*, especially when CNVs are present. However, many ambiguous results still provide useful and actionable information if they are fully explained and understood. Likewise, *1/*1 can also provide useful clinical guidance in supporting a standard course of treatment. However, they should also be considered with caution depending on the scope of the variants tested in a given report (i.e., more are usually better) and the population of origin of some patients (e.g., we should remember that knowledge bases are currently incomplete for those of non-European ancestry). Thus, follow-up testing with expanded assays, re-testing at some time in the future, or investing in continuously updated clinical interpretive reports may be useful in certain circumstances.

It is also important to maintain an awareness of developments in PGx as they occur in the coming years. Technological advancements are expected to enhance the utility of genomic data in the clinic, and the ever-expanding databases of clinical outcomes are likely to refine and expand the clinical decision support that is possible to deliver. Ultimately, PGx is a valuable tool in any clinician’s toolkit, and its reasonable use in refining patient-specific treatment plans has the potential to greatly improve the health and well-being of many psychiatric patients.

## Author Contributions

All authors contributed to research, manuscript writing, and revisions and have read and approved the submitted version.

## Funding

This work was supported by Coriell Life Sciences.

## Conflict of Interest Statement

Authors JJ, AP, and JS were employed by company Coriell Life Sciences. JJ and JS have equity interest in Coriell Life Sciences.
