# Supplementary material for: Consequences of CYP2D6 Copy-Number Variation for Pharmacogenomics in Psychiatry
Source: Front Psychiatry. 2019 Jun 20;10:432. doi: 10.3389/fpsyt.2019.00432 (PMC6595891; doi:10.3389/fpsyt.2019.00432)
Supplement: Supplementary file 1 [file DataSheet_1.docx]

**
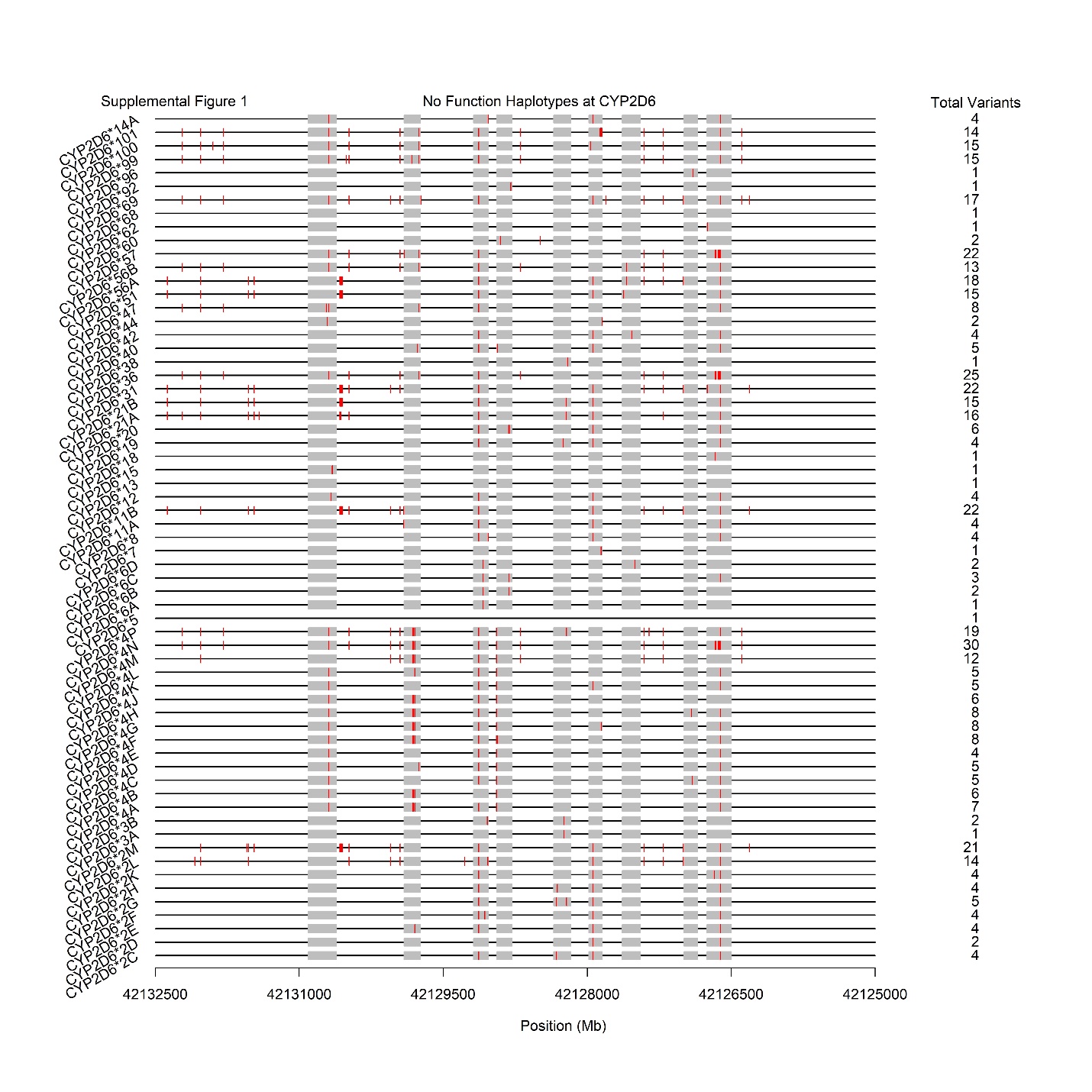
**

**Supplemental Figure 1:** Physical positions (red vertical lines) and total number of variants present on the 63 known CYP2D6 haplotypes (*alleles) predicted to produce non-functional enzymes upon transcription/translation.


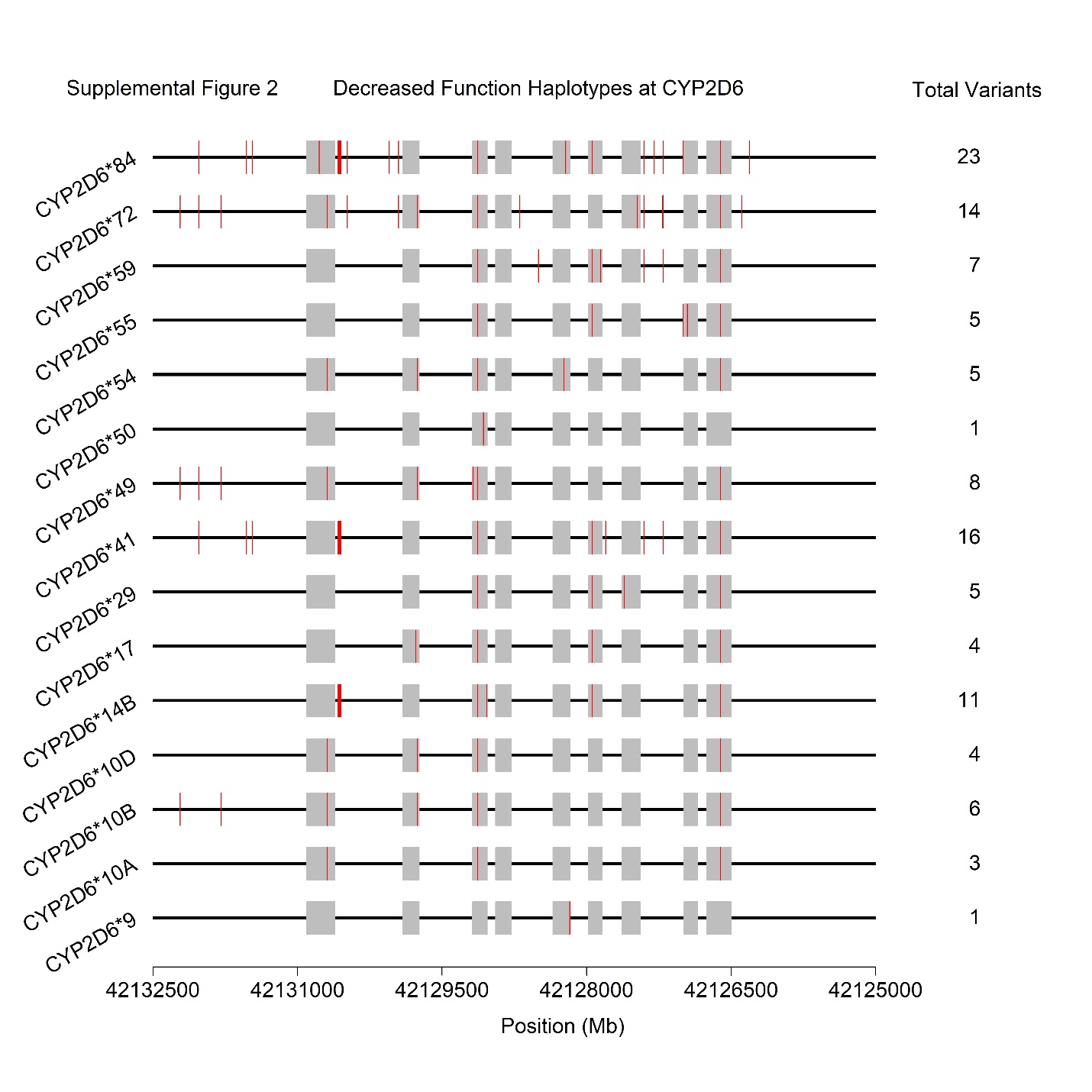


**Supplemental Figure 2:** Physical positions (red vertical lines) and total number of variants present on the 15 known CYP2D6 haplotypes (*alleles) predicted to produce enzymes with decreased function upon transcription/translation.


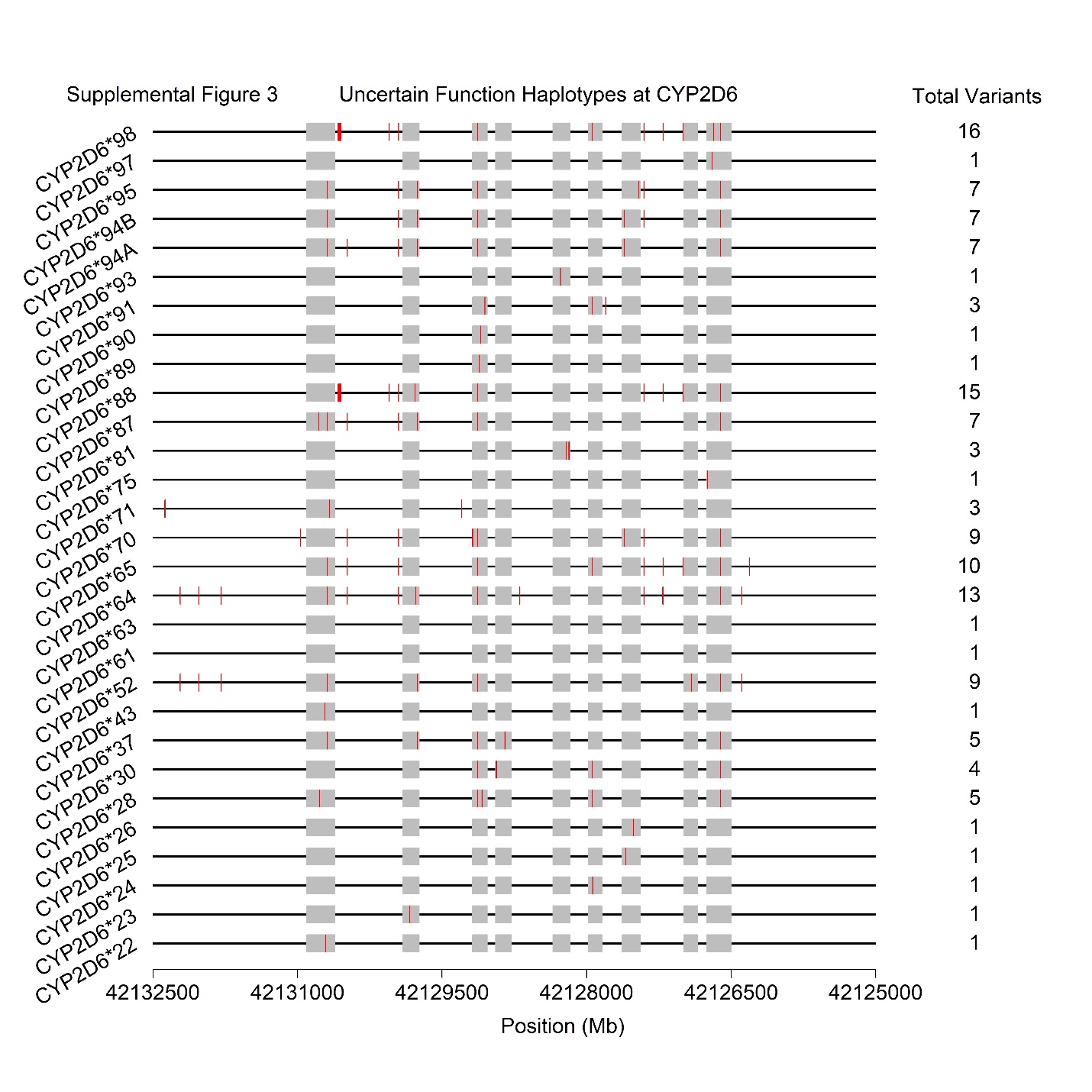


**Supplemental Figure 3:** Physical positions (red vertical lines) and total number of variants present on the 29 known CYP2D6 haplotypes (*alleles) that produce enzymes with uncertain functional characteristics (i.e. results and research findings are conflicting or inconclusive) upon transcription/translation.


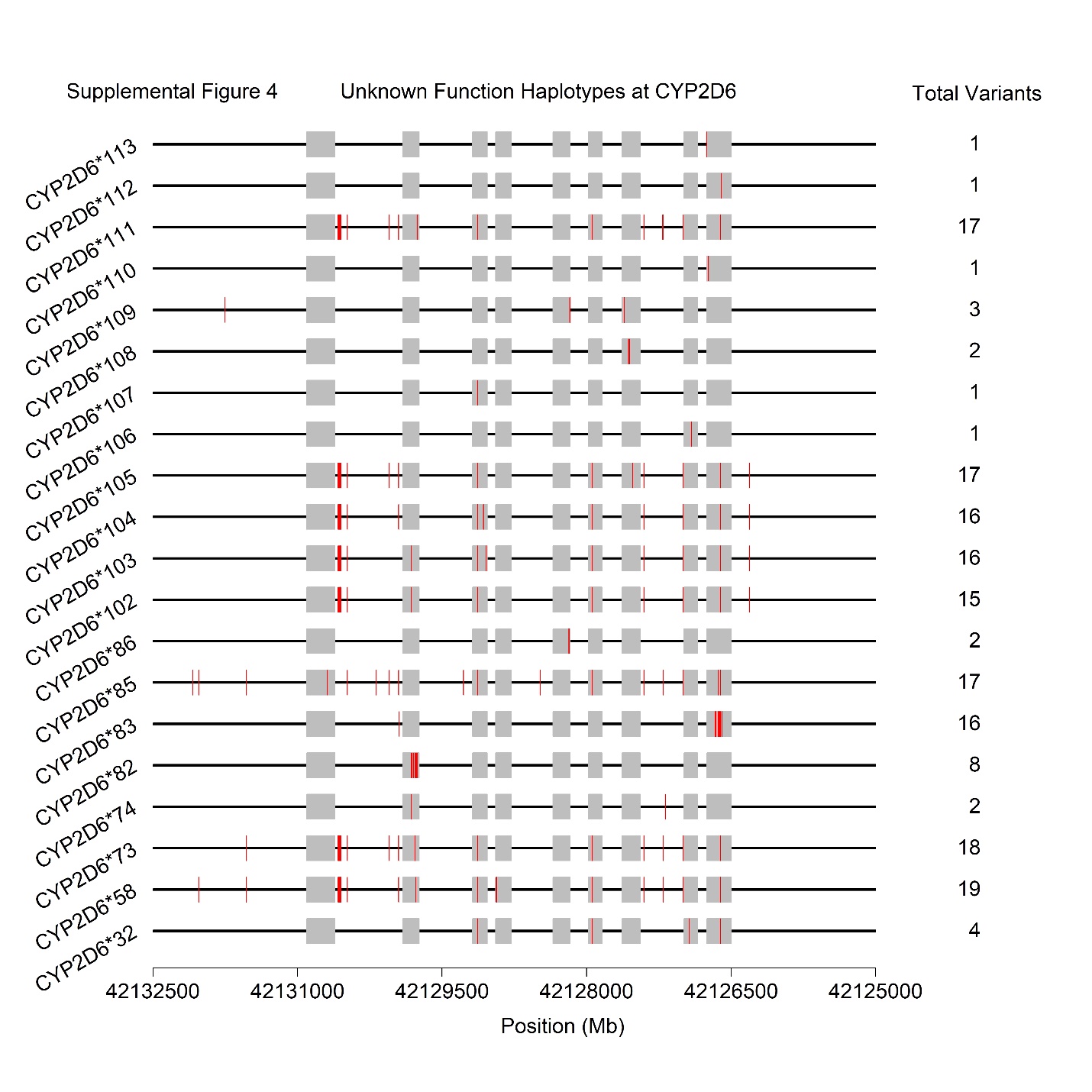


**Supplemental Figure 4:** Physical positions (red vertical lines) and total number of variants present on the 20 known CYP2D6 haplotypes (*alleles) that produce enzymes with unknown functional characteristics (i.e. variant combinations that are too rare to effectively interpret or which have not been adequately studied) upon transcription/translation.
